# P-1512. Partnering Imipenem/Relebactam (I/R) with Fosfomycin (FOS): Restoring Susceptibility Against Multi-Drug-Resistant *Pseudomonas aeruginosa*

**DOI:** 10.1093/ofid/ofae631.1681

**Published:** 2025-01-29

**Authors:** Scott A Becka, Elise T Zeiser, Maria F Mojica, Robert Bonomo, Gauri G Rao, Krisztina M Papp-Wallace

**Affiliations:** Cleveland VA Medical Center, Cleveland, Ohio; Cleveland VA Medical Center, Cleveland, Ohio; Case Western Reserve University, Cleveland, Ohio; Case Western, Cleveland, Ohio; USC Alfred E. Mann School of Pharmaceiti, Rancho Palos Verdes, California; Element-Iowa City, formerly JMI Laboratories, North Liberty, Iowa

## Abstract

**Background:**

*Pseudomonas aeruginosa* (*Pae*) is a significant pathogen causing up to 10% of hospital-acquired infections. Contributing to *Pae*’s multidrug-resistant (MDR) phenotype is the inducible *Pseudomonas*-derived cephalosporinase (PDC), low permeability outer membrane, multiple efflux systems, and loss of porins that yield resistant phenotypes. e.g. loss of OprD yields resistance to imipenem. Thus, the design of novel antibiotics or combination therapies that do not induce expression of *bla*_PDC_, are stable to PDC hydrolytic activity, bypass impermeability, and/or are not susceptible to efflux is essential for combating MDR *Pae* infections. Ceftazidime-avibactam (CZA), and imipenem/cilastatin/relebactam (I/R) were approved by the FDA for the treatment of infections caused by *Pae*; however, gaps in their efficacy against *Pae* remain. Previously, Winkler et al. analyzed a group of archived *Pae* and found that 20% of the isolates were resistant to CZA (PMID 25451057). By combining CZA with fosfomycin (FOS) and targeting multiple cell wall synthetic pathways, susceptibility was restored to most of the CZA resistant MDR *Pae* (PMID: 31099835). Based upon a similar mechanism of action, we propose that the combination of I/R+FOS will also restore susceptibility to I/R resistant *Pae*.

**Figure:**
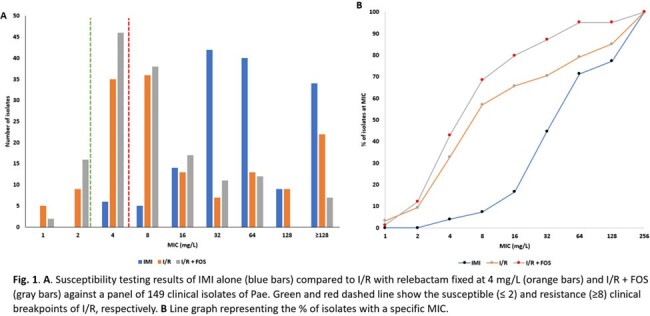

**Methods:**

149 isolates of *Pae* were used in this analysis. Minimum inhibitor concentrations (MICs) of IMI, I/R, and I/R+FOS against 150 *P. aeruginosa* isolates were determined following CLSI’s approved methods. To interpret the results better, genetic analysis of the resistomes was performed.

**Results:**

The addition of FOS was found to shift the I/R MICs towards the susceptible range (**Fig.1A**). Moreover, the isolates tested with I/R+FOS possessed overall lower MICs (MIC_50/90_ I/R 8/256 mg/L vs. MIC_50/90_ I/R+FOS 8/64 mg/L) (**Fig. 1B**). The isolates have diverse resistance mechanisms including KPC-producers (3/149; 4.5%), VIM-producers (19/150; 28.3%), and isolates that carry mutations in *ampR*, *ampD*, *oprD*, and/or *pbp* genes.

**Conclusion:**

I/R + FOS lowers MICs against MDR *Pae*. These results lead us to postulate that inhibiting MurA enzyme that catalyzes the first committed step in peptidoglycan synthesis with FOS adds to the potency of I/R even against VIM producers.

**Disclosures:**

**Robert Bonomo, MD**, Merck: Grant/Research Support|Shionogi: Grant/Research Support **Gauri G. Rao, PharmD, MS**, Merck: Grant/Research Support

